# Quantitative analysis of peri-intestinal lymph node metastasis using indocyanine green fluorescence imaging technology

**DOI:** 10.1097/MD.0000000000039240

**Published:** 2024-08-30

**Authors:** Weiyang Lin, Qi Li, Jie Sheng, Yibing Zhao, Wei Cui

**Affiliations:** a Ningbo Medical Center Lihuili Hospital, Ningbo University, Ningbo, China.

**Keywords:** colorectal cancer, indocyanine green, lymph node, malignant tumor metastasis, nanocarbon, near-infrared fluorescence

## Abstract

We evaluated the efficacy of indocyanine green fluorescence imaging compared to that of traditional nanocarbon dyes in assessing peri-intestinal lymph node metastasis in patients with colorectal cancer, which is a key prognostic factor. The relationship between indocyanine green fluorescence imaging and histopathological outcomes in patients with colon cancer has also been explored. A retrospective analysis was conducted on 30 patients with colon cancer (from May to October 2023) confirmed by surgical pathology. Tumors were marked with indocyanine green (ICG) or nanocarbon via colonoscopy 16 to 24 hours before surgery. Within 15 minutes after surgery, peri-intestinal lymph node fluorescence imaging and hematoxylin and eosin staining were used to assess the distribution of cancer foci. The correlation between cancer foci distribution, fluorescence intensity, and area under the receiver operating characteristic curve was measured. Among 243 metastatic lymph nodes from 30 patients, 18 were found. After the patients were divided into metastatic and nonmetastatic groups, significant differences in tumor differentiation and stage were noted (*P* < .001). The fluorescence intensity was strongly correlated with the presence and proportion of metastasis (area under the receiver operating characteristic curve = 0.931), whereas nanocarbon staining showed no significant correlation (*P* = .81). All *P* values were two-sided, with *P* < .05 indicating statistical significance. Lymph nodes with malignant intestinal tumor metastasis displayed weaker ICG fluorescence than did nonmetastatic nodes. Combining ICG and nanocarbon staining techniques enhances intraoperative lymph node dissection and postoperative analysis, indicating their potential utility in colorectal cancer surgery.

## 1. Introduction

Colorectal cancer is a common malignant tumor and the second leading cause of cancer-related death worldwide.^[1]^ Metastasis to the peri-intestinal lymph nodes is one of the reasons for poor prognosis.^[2]^ Traditional preoperative assessment methods such as imaging-based diagnostics are subjective and yield inconclusive results. Although there has been progress in relevant technologies, it remains challenging to accurately identify metastatic lymph nodes during surgery. In recent years, various intraoperative navigation techniques have been reported in the field of surgery, among which indocyanine green fluorescence imaging (ICG-FI) is a feasible option for observing intraoperative lymph node drainage and anastomotic perfusion. A prospective study involving 23 patients revealed that due to the lack of specific receptor targets, the fluorescence signal does not have strong specificity for detecting lymph node metastasis.^[3]^ Similarly, Nishigori et al observed metastases in 10% of ICG-fluorescent lymph nodes and 5.3% of nonfluorescent lymph nodes.^[4]^ However, owing to the small number of patients, lack of randomness, and short follow-up time, statistically meaningful results could not be obtained. This study aimed to compare the accuracy of ICG-FI staining with that of nanocarbon staining in diagnosing peri-intestinal lymph node metastasis during colorectal cancer surgery. By comparing its findings with pathological results, ICG-FI technology shows potential as a reliable tool for clinicians to guide surgical decision-making and develop individualized treatment plans, thereby improving the survival rate and quality of life of patients with colorectal cancer.

## 2. Research methods and materials

### 2.1. Research subjects

This prospective study was approved by the Ethics Consultation Committee of Ningbo Medical Center, Li Huili Hospital. Written informed consent was obtained from all the patients included in the study. All methods in this study were conducted in accordance with the relevant guidelines and regulations and were in compliance with the Helsinki Declaration. Informed consent was obtained from all participants and/or their legal guardians. Additionally, appropriate measures were taken to protect the privacy and rights of the participants, ensuring that the study did not subject them to unnecessary risks or inconveniences. From May 2023 to October 2023, patients treated at Ningbo Medical Center Li Huili Hospital were included if they met the following criteria: (1) had a diagnosis of colorectal cancer through laboratory tests, radiological examinations, and colonoscopy and required radical tumor resection surgery; (2) had available clinical data and routine pathological lymph node results; (3) voluntarily participated in the study and signed the informed consent form; and (4) were aged ≥18 and ≥85 years. The exclusion criteria were as follows: (1) history of colon surgery; (2) allergy or adverse reactions to ICG; (3) pregnancy or the need for emergency surgery; and (4) a maximum diameter of <1 mm for all dissected lymph nodes. Thirty patients, including 18 males and 12 females, were enrolled.

### 2.2. Preoperative and intraoperative procedures

**Figure 1.**
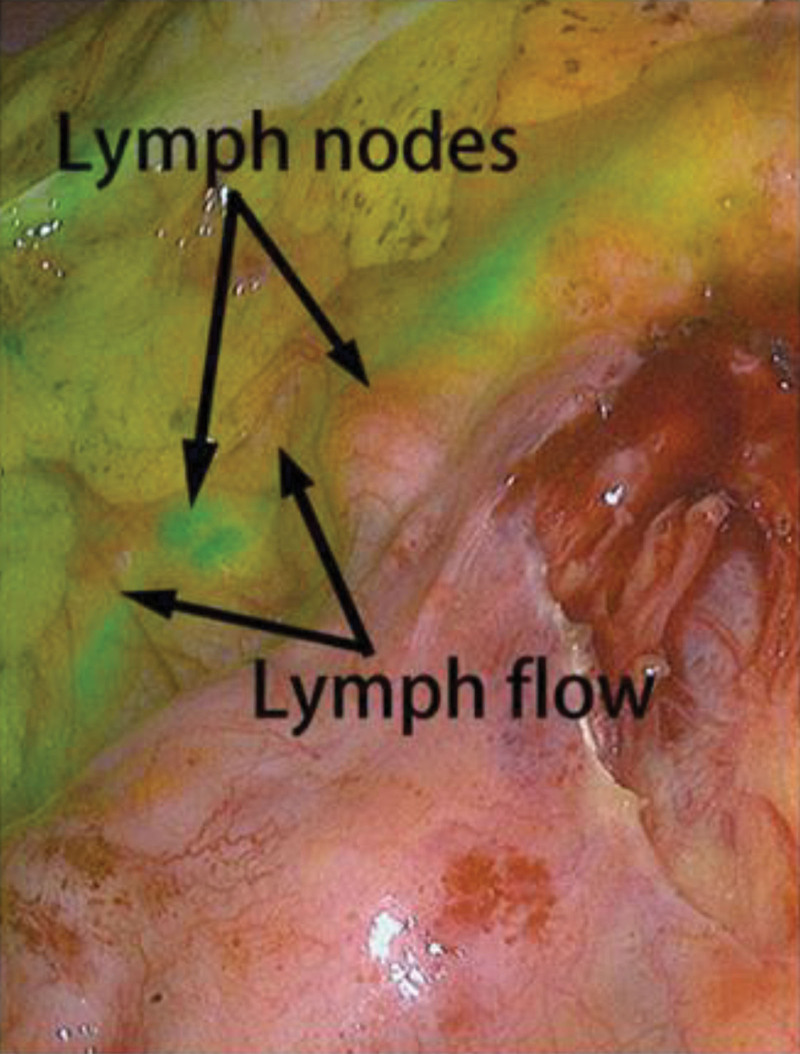
Intraoperative visualization of the lymph flow and lymph nodes.

Sixteen to twenty-four hours before surgery, indocyanine green (ICG) was administered via colonoscopy for tumor marking and localization. For each patient, 25 mg of powdered ICG was dissolved in 10.0 mL of sterile injection water. The injection was performed at 3 points on the normal intestinal wall, 1 cm distal to the tumor’s lower edge, with one injection point at the site and one on each side, 1 cm apart. A total of 1.0 mL of the solution was injected each time.^[5]^ The nanocarbons were simultaneously injected at the same injection site. A PC9001 near-infrared fluorescence imaging system (Stryker Corporation) was used to image the lymph nodes. During surgery, routine lymph node dissection was performed, and a PC9001 near-infrared fluorescence imaging system (Stryker Corporation) was used to observe the lymph flow and lymph nodes (Fig. 1).

### 2.3. Lymph node analysis

Lymph node dissection was completed within 15 minutes of surgical specimen removal. All lymph nodes in the specimen were dissected along the intestine, above and below the tumor, and their distance from the tumor margin was recorded. The surrounding connective tissue was removed as much as possible to minimize its negative effects on the imaging quality under the fluorescence lens. To ensure clarity in the fluorescence images, lymph nodes with a maximum diameter >0.1 cm were selected. The fluorescence lens was positioned to face the lymph nodes along its largest cross-sectional dimension and fixed with the mechanical arm of the device; then, the system was switched to fluorescence mode, and lymph node fluorescence photography was conducted under conditions simulating intra-abdominal photography. Subsequently, the dissected lymph nodes were numbered, fixed with formalin, and subjected to pathological analysis. A pathologist with >5 years of experience assessed whether the lymph nodes were metastatic. Furthermore, we processed the obtained lymph node images using Image Plus Pro 6.0.0.260 software to obtain data such as the average fluorescence intensity and optical density of the lymph nodes and recorded the information alongside the postoperative lymph node biopsy results.

### 2.4. Data analysis

Data analysis was performed using IBM Statistical Package for the Social Sciences Statistics for Windows version 26.0. Clinical patient data, including sex, age, body mass index, preoperative staging, and postoperative body temperature, were also collected. Patients were divided into lymph node metastasis and nonmetastasis groups, and the Mann–Whitney U test or Fisher exact test was used for data comparisons between the groups. For categorical data, such as pathological stage and histological type, the rank-sum test was used to evaluate differences between the groups. Additionally, to better understand these comparisons, we calculated the area under the receiver operating characteristic curve (AUC). To construct the receiver operating characteristic curve, sensitivity (true positive rate) was plotted against 1-specificity (false positive rate) across various threshold values of fluorescence intensity. The AUC was then calculated to quantify the overall discriminative ability of the test to differentiate between metastatic and nonmetastatic lymph nodes. Given the unbalanced distribution between the metastasis and nonmetastasis groups, we employed stratified sampling to ensure adequate representation and applied weighted adjustments in the statistical analysis to balance the influence of the smaller group. These methods help to provide a more accurate and unbiased evaluation of the data. Normally distributed data are expressed as the means and standard deviations, whereas nonnormally distributed data are expressed as medians and interquartile ranges. All *P* values were two-sided, and statistical significance was set at *P* < .05.

## 3. Results

To assess the effectiveness of ICG in identifying peri-intestinal lymph node metastasis, we conducted a comparative analysis of the clinical and tumor characteristics of patients with and without lymph node metastasis. This analysis can help elucidate the accuracy of ICG in localizing and identifying metastatic lymph nodes, thereby revealing the potential application value of this technology in colorectal cancer surgery. A total of 243 lymph node samples were collected from 30 patients. Among these samples, malignant tumor metastases were found in 18 lymph nodes of 9 patients (30%). Table 1 shows the clinical and pathological characteristics and surgical outcomes of the patients. The lymph node metastasis group included 3 males and 6 females, with an average age of 63.26 years and an average body mass index of 24.45 kg/m^2^. The maximum tumor diameter in this group was significantly greater than that in the nonmetastasis group (*P* = .018), and the lymph node metastasis group had a significantly greater degree of tumor differentiation and was significantly more likely to have later pathological tumor stages than the nonmetastasis group (*P* < .001). There were no statistically significant differences in other factors between the 2 groups.

**Table 1:** Clinical pathological features and surgical outcomes of the patient groups.

	Metastatic lymph node group ^(n=9)^	Nonmetastatic lymph node group ^(n=21)^	*P*-value
Age (x ± s, year)	63.56 ± 13.44	68.95 ± 5.95	.276
Body mass index (x ± s, kg/m^2^)	24.45 ± 2.88	23.16 ± 2.48	.248
Sex			.055
Male	3	15	
Female	6	6	
CEA	2.2	2.1	.23
CA19-9	14.2	8.5	.06
Max-axis (mm) (x ± s, cm)	4.32 ± 1.90	2.95 ± 1.09	.018
Differentiation			.035
Poorly	5	4	
Moderately	4	14	
Well	0	3	
Pathological staging			0
I	0	2	
II	3	19	
III	6	0	
Histological type			.346
Ulcerative	6	10	
Polypoid	3	11	
Number of dissected LNs*	10 (8–17)	7 (5–8)	.005
Number of Metastatic LNs*	1.5 (1–5)	_-_	
Operative time (hours)	3. 16 ± 0.18	3.28 ± 0.90	.304
Localization-surgery end time (hours)	19.67	22.75	.375
Blood loss (mL)*	50 (25–50)	50 (25–50)	.781

* Numerical data are presented as medians. Values in parentheses are Interquartile ranges (IQRs); first quartile–third quartile.

**Figure 2.**
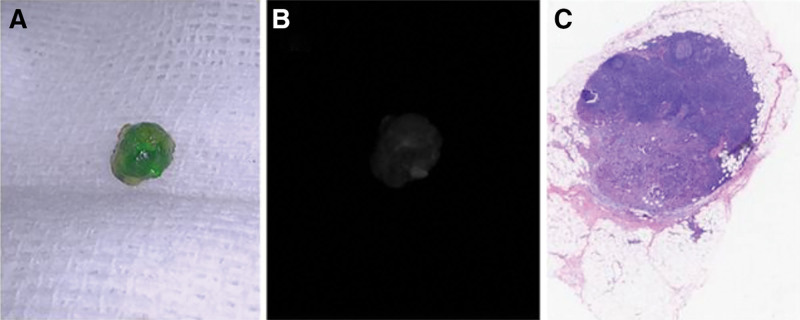
(A) Photograph of a metastatic lymph node stained with ICG under the visible light mode of the near-infrared photography system (lymph node number: 04-11). (B) Photograph of metastatic lymph node 04-11 under fluorescence mode, showing weak fluorescence intensity. (C) HE staining of metastatic lymph nodes 04-11 (×20 magnification). ICG = indocyanine green.

**Figure 3.**
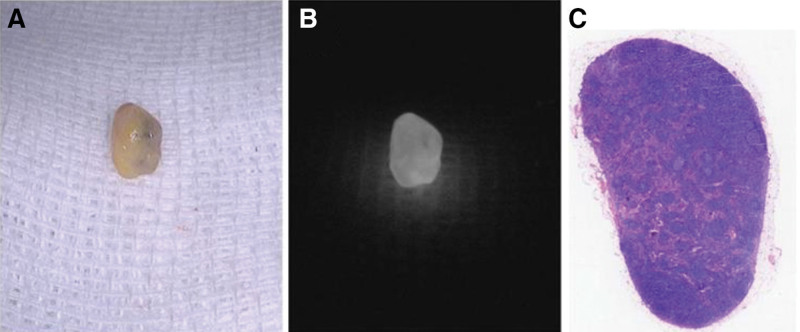
(A) Photograph of a nonmetastatic lymph node stained with ICG under the visible light mode of the near-infrared photography system (lymph node number: 04-14). (B) Photograph of nonmetastatic lymph node 04-14 under fluorescence mode, showing significantly stronger fluorescence intensity than that observed for metastatic lymph nodes. (C) HE staining of nonmetastatic lymph node 04-14 (×20 magnification). ICG = indocyanine green.

**Figure 4.**
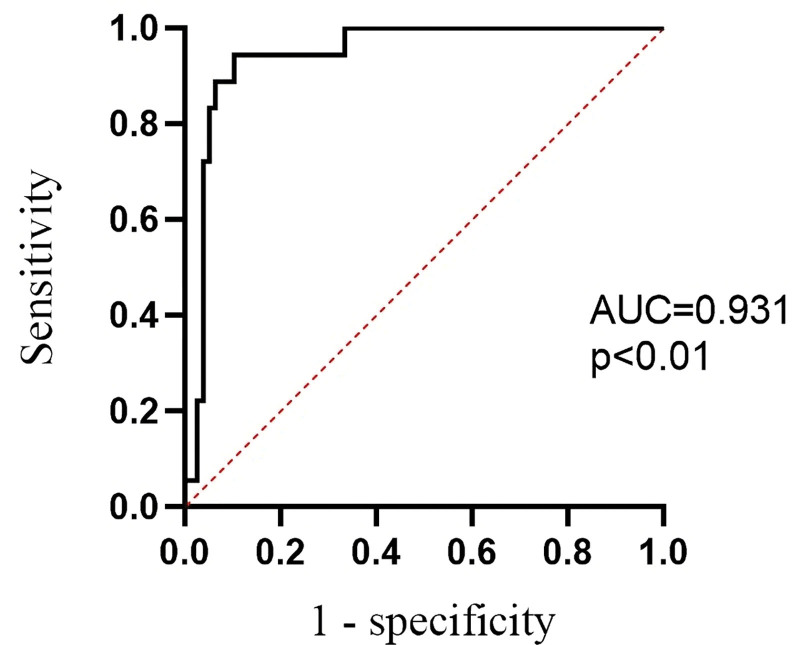
ROC curve of the average fluorescence intensity of ICG-stained lymph nodes. ICG = indocyanine green, ROC curve = receiver operating characteristic curve.

Next, we evaluated the ability of ICG-FI to differentiate between metastatic and nonmetastatic lymph nodes within the lymph node metastasis group. In this study, 96 lymph nodes were obtained from 9 patients in the lymph node metastasis group, of which 18 (18.8%) had metastatic disease (Figs. 2 and 3). The lymph nodes were divided into metastatic and normal lymph nodes, and their clinical characteristics are shown in Table 3. The average fluorescence intensity of the metastatic lymph nodes (25.23 ± 13.36) was significantly lower than that of the nonmetastatic lymph nodes (75.84 ± 29.44, *P* < .001). The median number of dissected lymph nodes was 10 (interquartile range [IQR]: 8–17), and the median number of metastatic lymph nodes was 1.5 (IQR: 1–5). Table 2 provides a detailed description of all the metastatic lymph nodes. The metastatic lymph nodes had significantly larger maximum diameters (*P* = .03) and were significantly closer to the tumor margin than the nonmetastatic lymph nodes (*P* < .001). The median percentage of the tumor metastasis area out of the total area among these metastatic lymph nodes was 45% (IQR: 30–82.5%). The fluorescence intensity of the lymph nodes significantly correlated with the occurrence of metastasis (*P* < .001) and the proportion of metastasis (*P* < .001). The AUC was 0.931 (*P* < .01) (Fig. 4). However, there was no significant difference in the percentage of patients with positive nanocarbon staining between the 2 lymph node groups (*P* = .81).

**Table 2:** Details of metastatic lymph nodes.

Case	LN number	Max-axis (mm)	Fluorescence intensity	Nanocarbon staining	Occupatio n	% occupation (%)
1	01-04	8	20.39	Absent	Incomplete	45
3	03-01	5	25.46	Present	Incomplete	30
5	05-11	5	32.11	Absent	Incomplete	30
7	07-08	10	76.41	Present	Incomplete	15
7	07-09	8	23.91	Present	Incomplete	30
7	07-10	8	22.07	Absent	Incomplete	25
7	07-11	3	23.65	Present	Incomplete	25
7	07-13	3	16.53	Absent	Complete	100
7	07-15	8	24.8	Absent	Incomplete	45
14	14-05	2	17.92	Absent	Complete	100
14	14-09	2	18.42	Absent	Incomplete	85
21	21-01	4	28.36	Present	Incomplete	45
18	18-01	6	21.83	Present	Incomplete	45
18	18-05	15	15.06	Present	Incomplete	75
18	18-06	12	27.18	Absent	Incomplete	45
26	26-08	6	15.46	Absent	Complete	100
26	26-09	3	6.26	Absent	Complete	100
28	28-02	8	65.29	Present	Incomplete	15

**Table 3:** Clinical characteristics of lymph nodes in patients in the metastatic lymph node group.

	Metastatic lymph (n = 18)	Nonmetastatic lymph (n = 78)	*P* value
Average fluorescence intensity	25.23 ± 13.36	75.84 ± 29.44	0
Maximum fluorescence intensity	70.31 ± 13.36	120.35 ± 28.16	0
Minimum fluorescence intensity	3.70 ± 2.74	13.73 ± 11.72	0
IOD	6. 1 ± 0.36	6.4 ± 0.37	0
Lymph node metastasis percentage* (%)	45 (30–82.5)	–	0
Nanocarbon staining			.81
Present	8	52	
Absent	10	26	
Max-axis (mm)	6.44 ± 3.55	4.59 ± 2.89	.03
Distance to tumor margin (mm)	11.6 ± 13.18	24.62 ± 8.08	0

* Numerical data are presented as median. Values in parentheses are IQR; first quartile to third quartile.

## 4. Discussion

Intraoperative ICG fluorescence imaging technology has been widely used in various surgical procedures, mainly for intraoperative lymph node tracing and monitoring of intestinal anastomosis blood flow in patients with diseases such as colorectal, liver, and breast cancer.^[6–8]^ We focused our research on the quantitative relationship between lymph node fluorescence intensity and metastatic status and introduced a novel monitoring scheme that can intuitively indicate whether lymph nodes are metastatic and assess the extent of metastasis during colorectal cancer surgery. In this study, we performed standard radical resection for colorectal cancer in all patients, as specified in the 2018 NCCN guidelines for colorectal cancer.^[1]^ Intraoperative lymph node imaging was unrelated to the surgical method, and all lymph node data collection was completed within 15 minutes after surgery to ensure accurate results. These findings not only enhance the clinical application value of ICG fluorescence imaging but also provide new insights into the diagnosis and treatment of colorectal cancer.

In this study, most lymph nodes showed varying degrees of fluorescence through near-infrared photographic lenses, with a small portion showing almost no fluorescence. The nonmetastatic lymph nodes displayed uniform and strong fluorescence (Fig. 3), whereas those with metastasis showed relatively weak fluorescence (Fig. 2). Lymph nodes completely occupied by cancer cells emitted almost no fluorescence. Quantitatively, the average fluorescence intensity of the metastatic lymph nodes was 25.23 ± 13.36, which was significantly lower than that of the nonmetastatic lymph nodes (75.84 ± 29.44; *P* < .001). The AUC was 0.931, indicating that indocyanine green fluorescence was highly effective in predicting lymph node metastasis. According to Sato Y and others, this may be related to different degrees of blockage of lymphatic vessels by tumor cells, resulting in lymph flow obstruction.^[9]^ Other studies have shown that tumor cell metastasis can affect the microenvironment around the lymph nodes, altering blood and lymphatic flow within them,^[10,11]^ making it difficult for ICG to flow into the metastasized lymph nodes and affecting the distribution of ICG. Additionally, the metabolic environment within metastatic lymph nodes, characterized by hypoxia or inflammation,^[12]^ might also interfere with the fluorescence characteristics of ICG. These differences suggest that lymph nodes with weak fluorescence signals might contain malignant tumor metastases and should be carefully dissected postoperatively to provide clinical surgeons with a more intuitive and accurate basis for lymph node discrimination and intraoperative staging during surgery.

Notably, although the imaging findings of most lymph nodes conformed to this pattern, some lymph nodes did not follow this trend. This deviation from expectations may be caused by various factors. First, there may be micrometastases in the lymph nodes, meaning that the diameter of the small deposits of malignant cells inside them is <2 mm.^[13]^ For example, metastatic lymph nodes numbered 07-08 and 28-02 showed stronger fluorescence, and through HE staining, 15% of the metastatic lymph nodes were found to be metastatic (Table 3). The number of malignant tumor cells in these lymph nodes may not have reached a sufficient level to affect the ICG distribution of ICG. Second, according to multiple studies by Lee and Hüyük, lymph node micrometastases are rarely detected by conventional pathology but can be detected using techniques such as immunohistochemistry.^[14,15]^ Therefore, it can be assumed that some of the lymph nodes confirmed as nonmetastatic by HE staining may have contained micrometastases. Even 2 lymph nodes with almost identical clinical characteristics may have different fluorescence intensities. Finally, if the sectioned surface of the lymph node did not show metastasis, the specimen was diagnosed as negative. The presence of these phenomena suggests that the specificity of ICG staining remains unsatisfactory. Regardless of the fluorescence intensity of the lymph nodes, thorough dissection of the lymph nodes should be performed during surgery.

Nanocarbons, biocompatible dyes, have been widely used in tumor surgery to image lymph nodes.^[16,17]^ Compared to other dyes, including ICG, nanocarbons have better in vivo stability and persistence.^[18]^ According to Xie et al, the use of nanocarbon staining for lymph nodes is unrelated to the presence of malignant tumor cell metastases.^[19]^ In our study, statistical analysis revealed a *P* value of .81, which further confirmed that there was no significant association between nanocarbon staining and metastasis status. This indicates that although nanocarbon technology can effectively image lymph nodes, it does not help in differentiating benign from malignant lymph nodes or in assessing the presence of micrometastases within them. Furthermore, we explored the relationship between nanocarbon and ICG staining. We observed a greater proportion of lymph nodes that produced fluorescence after ICG staining (100%) than after nanocarbon staining (64.8%). This suggests that ICG, as a dye, has greater penetration in lymphatic tissues^[20]^ and aids in the optical recognition of lymph nodes. Additionally, the staining results of the 2 dyes were not significantly correlated (*R* = 0.02, *P* = .829), indicating that they may fluoresce under different mechanisms in lymph node imaging and should be used based on specific clinical needs and expected outcomes. Considering the superiority of nanocarbon staining for lymph node localization and the potential value of ICG in identifying lymphatic flow, we believe that both staining techniques should be used complementarily in clinical practice. In future research, the combined use of these 2 staining methods could provide new approaches for more precise imaging and assessment of lymph nodes.

Nevertheless, this study had certain limitations. The small sample size limits the reliability and generalizability of the results. Evaluating lymph node metastasis from such a limited perspective might result in missing more subtle metastases, potentially affecting experimental outcomes. In response, we will explore new methods in future experiments and expand the sample size to validate the conclusions of this study.

In conclusion, we found that the fluorescence emitted by lymph nodes with pathological intestinal malignant tumor metastasis after ICG staining was generally weaker than that emitted by nonmetastatic lymph nodes. This suggests that lymph nodes with weaker fluorescence intensity require meticulous dissection and pathological analysis during surgery. Additionally, we observed that nanocarbon agents can stain lymph nodes indiscriminately, indicating that different intraoperative staining techniques can complement each other and enhance surgical outcomes. More importantly, these findings not only have direct application value in colorectal cancer surgery but also may impact other areas, such as improving surgical techniques and increasing surgical safety and efficiency. Future research should explore the application of these techniques to a broader range of tumor types and optimize lymph node imaging technology to improve the accuracy of identifying tumor metastasis. Furthermore, it is necessary to comprehensively assess the impact of these staining techniques on the long-term prognosis of patients to provide additional guidance and insights for clinical practice.

## Author contributions

**Conceptualization:** Weiyang Lin.

**Data curation:** Weiyang Lin, Qi Li, Yibing Zhao.

**Formal analysis:** Weiyang Lin.

**Funding acquisition:** Wei Cui.

**Investigation:** Qi Li, Jie Sheng, Wei Cui.

**Methodology:** Weiyang Lin, Jie Sheng, Yibing Zhao, Wei Cui.

**Project administration:** Weiyang Lin, Wei Cui.

**Resources:** Qi Li.

**Validation:** Yibing Zhao, Wei Cui.

**Visualization:** Yibing Zhao.

**Writing – original draft:** Weiyang Lin.

**Writing – review & editing:** Weiyang Lin, Wei Cui.

## References

[1] Benson AB Venook AP Al-Hawary MM. NCCN guidelines insights: colon cancer, version 2.2018. J Natl Compr Canc Netw. 2018;16:359–69. 29632055 10.6004/jnccn.2018.0021 PMC10184502

[2] Lee YJ Huh JW Shin JK. Risk factors for lymph node metastasis in early colon cancer. Int J Colorectal Dis. 2020;35:1607–13. 32447479 10.1007/s00384-020-03618-7

[3] Staniloaie D Budin C Ilco A. In vivo sentinel lymph node detection with indocyanine green in colorectal cancer. Maedica. 2022;17:264–70. 36032598 10.26574/maedica.2022.17.2.264 PMC9375885

[4] Nishigori N Koyama F Nakagawa T. Visualization of lymph/blood flow in laparoscopic colorectal cancer surgery by ICG fluorescence imaging (Lap-IGFI). Ann Surg Oncol. 2016;23(Suppl 2):S266–74. 25801355 10.1245/s10434-015-4509-0

[5] Ahn HM Son GM Lee IY. Optimal ICG dosage of preoperative colonoscopic tattooing for fluorescence-guided laparoscopic colorectal surgery. Surg Endosc. 2022;36:1152–63. 33638107 10.1007/s00464-021-08382-5 PMC8758609

[6] Watanabe J Ota M Suwa Y Ishibe A Masui H Nagahori K. Real-time indocyanine green fluorescence imaging-guided complete mesocolic excision in laparoscopic flexural colon cancer surgery. Dis Colon Rectum. 2016;59:701–5. 27270525 10.1097/DCR.0000000000000608

[7] Watanabe J Ota M Suwa Y Ishibe A Masui H Nagahori K. Evaluation of lymph flow patterns in splenic flexural colon cancers using laparoscopic real-time indocyanine green fluorescence imaging. Int J Colorectal Dis. 2017;32:201–7. 27695977 10.1007/s00384-016-2669-4

[8] Lucas K Melling N Giannou AD. Lymphatic mapping in colon cancer depending on injection time and tracing agent: a systematic review and meta-analysis of prospective designed studies. Cancers. 2023;15:3196. 37370806 10.3390/cancers15123196 PMC10296374

[9] Sato Y Satoyoshi T Okita K. Snapshots of lymphatic pathways in colorectal cancer surgery using near-infrared fluorescence, in vivo and ex vivo. Eur J Surg Oncol. 2021;47:3130–6. 34373159 10.1016/j.ejso.2021.07.025

[10] Brown M Assen FP Leithner A. Lymph node blood vessels provide exit routes for metastatic tumor cell dissemination in mice. Science. 2018;359:1408–11. 29567714 10.1126/science.aal3662

[11] Hoshida T Isaka N Hagendoorn J. Imaging steps of lymphatic metastasis reveals that vascular endothelial growth factor-C increases metastasis by increasing delivery of cancer cells to lymph nodes: therapeutic implications. Cancer Res. 2006;66:8065–75. 16912183 10.1158/0008-5472.CAN-06-1392

[12] Penet MF Pathak AP Raman V Ballesteros P Artemov D Bhujwalla ZM. Noninvasive multiparametric imaging of metastasis-permissive microenvironments in a human prostate cancer xenograft. Cancer Res. 2009;69:8822–9. 19861534 10.1158/0008-5472.CAN-09-1782 PMC2783669

[13] Zhou Y Zhang GJ Wang J Zheng K-Y Fu W. Current status of lymph node micrometastasis in gastric cancer. Oncotarget. 2017;8:51963–9. 28881703 10.18632/oncotarget.17495 PMC5584304

[14] Lee CM Park SS Kim JH. Current status and scope of lymph node micrometastasis in gastric cancer. J Gastric Cancer. 2015;15:1–9. 25861517 10.5230/jgc.2015.15.1.1 PMC4389091

[15] Hüyük M Fiocco M Postmus PE Cohen D von der Thüsen JH. Systematic review and meta-analysis of the prognostic impact of lymph node micrometastasis and isolated tumour cells in patients with stage I–IIIA non-small cell lung cancer. Histopathology. 2023;82:650–63. 36282087 10.1111/his.14831

[16] Yan X Zeng R Ma Z. The utility of sentinel lymph node biopsy in papillary thyroid carcinoma with occult lymph nodes. PLoS One. 2015;10:e0129304. 26046782 10.1371/journal.pone.0129304 PMC4457868

[17] Yang SX Wei WS Jiang QH. Analysis of 246 sentinel lymph node biopsies of patients with clinical primary breast cancer by application of carbon nanoparticle suspension. J Obstet Gynaecol Res. 2018;44:1150–7. 29673015 10.1111/jog.13635

[18] Tian Y Lin Y Guo H. Safety and efficacy of carbon nanoparticle suspension injection and indocyanine green tracer-guided lymph node dissection during robotic distal gastrectomy in patients with gastric cancer. Surg Endosc. 2022;36:3209–16. 34254184 10.1007/s00464-021-08630-8 PMC9001219

[19] Ya X Qian W Huiqing L. Role of carbon nanoparticle suspension in sentinel lymph node biopsy for early-stage cervical cancer: a prospective study. BJOG. 2021;128:890–8. 32930483 10.1111/1471-0528.16504

[20] Brouwer OR Buckle T Vermeeren L. Comparing the hybrid fluorescent-radioactive tracer indocyanine green-99mTc-nanocolloid with 99mTc-nanocolloid for sentinel node identification: a validation study using lymphoscintigraphy and SPECT/CT. J Nucl Med. 2012;53:1034–40. 22645297 10.2967/jnumed.112.103127

